# Incidence and predictors of Uveitis in juvenile idiopathic arthritis in a Nordic long-term cohort study

**DOI:** 10.1186/s12969-017-0195-8

**Published:** 2017-08-18

**Authors:** Ellen Nordal, Veronika Rypdal, Terje Christoffersen, Kristiina Aalto, Lillemor Berntson, Anders Fasth, Troels Herlin, Susan Nielsen, Suvi Peltoniemi, Bjørn Straume, Marek Zak, Marite Rygg, for the Nordic Study Group of Pediatric Rheumatology (NoSPeR)

**Affiliations:** 1 Department of Pediatrics, University Hospital of North Norway, and Department of Clinical Medicine, UiT the Arctic University of Norway, Tromsø, Norway; 2 Department of Ophtalmology, University Hospital of North Norway, and Department of Clinical Medicine, UiT the Arctic University of Norway, Tromsø, Norway; 3 Pediatric Rheumatology Clinic, Hospital for Children and Adolescents, University of Helsinki, Helsinki, Finland; 4 Department of Women’s and Children’s Health, Uppsala University, Uppsala, Sweden; 5 Department of Pediatrics, Institute of Clinical Sciences, Sahlgrenska Academy, University of Gothenburg, Gothenburg, Sweden; 6 Department of Pediatrics, Aarhus University Hospital, Aarhus, Denmark; 7 Pediatric Rheumatology Clinic, Rigshospitalet Copenhagen University Hospital, Copenhagen, Denmark; 8 Department of Community Medicine, UiT the Arctic University of Norway, Tromsø, Norway; 9 Department of Clinical and Molecular Medicine, NTNU - Norwegian University of Science and Technology, and Department of Pediatrics, St. Olavs Hospital, Trondheim, Norway

**Keywords:** Juvenile idiopathic arthritis, Uveitis, Epidemiology, Biomarkers, Histones, Antinuclear antibody

## Abstract

**Background:**

The incidence of uveitis associated with juvenile idiopathic arthritis (JIA) varies around the world. Our aim was to investigate the incidence and predictors of uveitis in a Nordic population-based cohort.

**Methods:**

Consecutive JIA cases from defined geographical areas in Denmark, Finland, Sweden and Norway with disease onset between January 1997 to June 2000 were followed for median 98 months in this prospective longitudinal cohort study. Potential clinical and immunological predictors of uveitis were identified with logistic regression analysis.

**Results:**

Uveitis occurred in 89 (20.5%) of the 435 children with regular ophtalmologic follow-up among the 500 included. Chronic asymptomatic uveitis developed in 80 and acute symptomatic uveitis in 9 children. Uveitis developed at a median interval of 0.8 (range − 4.7 to 9.4) years after onset of arthritis. Predictors of uveitis were age < 7 years at JIA onset (Odds ratio (OR) 2.1, 95% confidence interval (CI) 1.3 to 3.5), presence of antihistone antibodies (AHA) > 15 U/ml (OR 4.8 (1.8 to 13.4)) and antinuclear antibodies (ANA) (OR 2.4 (1.5 to 4.0)). Mean combined IgM/IgG AHA was significantly higher in the uveitis group (19.2 U/ml) than in the non-uveitis group (10.2 U/ml) (*p* = 0.002). Young age at JIA onset predicted uveitis in girls (*p* < 0.001), but not in boys (*p* = 0.390).

**Conclusion:**

Early-onset arthritis and presence of AHA in girls, as well as presence of ANA in both genders, were significant predictors of chronic uveitis. The high incidence of uveitis in this long-term Nordic JIA cohort may have severe implications in a lifelong perspective.

## Background

Uveitis is the most common extra-articular manifestation of juvenile idiopathic arthritis (JIA). This sight-threatening condition contributes significantly to the disease burden of JIA [1, 2]. Chronic insidious uveitis in JIA is often asymptomatic, and it is a major challenge to diagnose the condition as early as possible for prompt and adequate treatment [3, 4]. The diagnosis may be obvious in cases with acute uveitis [5], but the challenge remains to provide optimal screening programs and treatment to minimize long-term complications in all forms of JIA-associated uveitis. The reported prevalence of uveitis in JIA varies highly, and ethnic and geographic differences seem to exist. Recent publications show a prevalence of 8–25% in most Caucasian populations [6, 7], while some studies from other continents show lower percentage of uveitis, 1 to 7% [8, 9]. Young age at onset of arthritis and presence of ANA were predictors of uveitis in most studies [3, 10–12]. In some larger cohort studies and registries, oligoarticular onset category and female gender were also statistically significant predictors of uveitis, but with less clinical impact [3, 13].

Histones are subcomponents of chromatin where these basic DNA-binding proteins are found in highly organized nucleosomal particles [14]. Antihistone antibodies (AHA) are among the ANA subtypes identified in subsets of children with JIA, while other subtypes associated with connective tissue diseases such as anti-DNA, anti-RNP, anti-SSA and anti-SSB antibodies are rarely found [15, 16]. AHA were reported to be associated with early-onset JIA, oligoarthritis and uveitis [17]. We showed previously that AHA is a significant predictor of chronic uveitis in a Norwegian JIA cohort [11]. Whether this association is merely an epiphenomenon is not yet known, although new interest for histones in autoimmune diseases has emerged through recent epigenetic findings [18, 19].

A longitudinal cohort study of JIA in a population-based setting in the Nordic countries started in 1997 and is still ongoing [20, 21]. The ophthalmologic data of a follow-up 8 years after disease onset of this Nordic JIA cohort were analyzed in the present study. The main objective was to describe the incidence and the predictors of uveitis in JIA during the first decade after disease onset. We also wanted to extend the previous Norwegian pilot study to the Nordic JIA cohort regarding the presence of histone antibodies in patients with JIA with and without uveitis, respectively.

## Methods

### Patients

In this longitudinal study, children with long-term ophthalmologic follow-up were selected from a prospective multicentre Nordic JIA cohort. The original multicentre cohort consisted of consecutive children with newly diagnosed JIA and disease onset between January 1, 1997 to June 30, 2000 from defined geographical areas of Denmark, Finland, Norway and Sweden aiming for the cohort to be population-based [20]. Clinical information including family history, assessment of disease activity, self-reported questionnaires and information from ophthalmology visits was registered per protocol at baseline and at 1 to 3 year intervals. Detailed description of the methods of patient accrual and data collection has been published previously [21].

At the final study visit, at least 7.0 years (median 98 months, range 84–147 months) after disease onset, extended data including information from the most recent ophthalmologic visit was collected. The interval of ophthalmologic visits was every 2–3 months the first 2 years, then longer intervals according to time after disease onset and JIA category. The intervals were in line with the guidelines for uveitis screening at each center, based on international uveitis screening guidelines. Uveitis was registered as present and active, when the ophthalmologist prescribed treatment for inflammation in the uvea. Uveitis was classified according to the recommendations by the Standardization of Uveitis Nomenclature (SUN) working group [22]. Uveitis was classified as “acute”; symptomatic with acute onset, sometimes recurrent course but of limited duration, or “chronic”; insidious onset, mainly asymptomatic with chronic recurrent or persistent course. Collected data also included locally analyzed HLA-B27, antinuclear antibodies (ANA) and rheumatoid factor (RF), and a positive result of the latter two had to be confirmed, at least 3 months apart. Each physician interpreted the results of ANA and RF analysis as positive or negative according to the reference values of their local laboratory; the cut-off value for a positive test was ≥ 1/80 in Tromsø, Trondheim and parts of Sweden, ≥ 1/320 in Finland, and ≥ 1/160 in Copenhagen, Århus and some parts of Sweden. ANA was measured using immunofluorescence on HEp-2 cells. Serum was taken at a median of 4 (1st quartile of 2, 3rd quartile of 7) months after the disease was diagnosed, frozen and stored at −70 °C in aliquots to avoid repeated freeze-thaw cycles. AHA were detected with Varelisa Histone Antibodies Enzyme Induced Assay (EIA) kit for antihistone IgM/IgG antibodies using a combined IgM/IgG conjugate (Phadia Diagnostics, Freiburg, Germany) at UIT The Arctic University of Norway, Tromsø, Norway. Tests were run according to the manufacturer’s instructions [23]. According to the kit instructions a level ≥ 30 U/ml is considered positive, 15–29 U/ml borderline, and < 15 U/ml negative, based on a test population of 432 healthy adults, normative values are not given for children. In the present study, we investigated ≥ 15 U/ml as a positive threshold based on our previous study reporting a mean concentration of 4.3 U/ml in 58 healthy children utilizing the same Varelisa Histone EIA-kit [11]. The presence of AHA in a Norwegian JIA cohort, also including the Norwegian subsets of this Nordic JIA cohort, has previously been reported and was therefore not included here [11]. No sera were available from the Finnish participants, and subsequently analyses of the AHA were performed only in the Swedish and Danish sera.

Approval from medical research ethical committees and data protection authorities was granted according to the regulations of each participating country, in Norway from Regional Committee for Medical and Health Research Ethics NORD, number 53/96*.* Oral informed assent was obtained from all children. Written informed consent was obtained from parents of children aged < 16 years and from the children if aged ≥ 16 years.

### Statistical analyses

The study report followed the STROBE guidelines [24]. Statistical analyses were performed using the STATA version 14 software (STATA Corp., College Station, TX, USA). Descriptive statistics were used to summarize clinical characteristics of the population and disease activity measures. The chi-square test was used as appropriate for comparison of categorical variables. The Student’s *t*-test was used to compare means in continuous outcomes between groups, and Mann-Whitney rank sum test for comparison of medians for skewed data. A logistic regression analysis of baseline characteristics was performed to calculate odds ratio for developing uveitis. The *p*-value <0.05 was considered significant.

## Results

Regular ophthalmologic follow-up for more than 7 years was available in 435 (87.0%) of the 500 children included in the prospective multi-centre Nordic JIA-cohort; 60 children were lost to follow-up, and in 5 children long-term ophthalmological data were missing. Baseline clinical characteristics of the group with or without long-term follow-up were mostly similar and have previously been published [21].

### Incidence

Uveitis developed in 89 (20.5%) of the 435 children. Clinical characteristics at baseline according to the presence of acute and chronic uveitis is shown in Table 1. Among the 9 children with acute uveitis, 8 had enthesitis-related arthritis (ERA), and 7 were HLA-B27 positive. Chronic insidious uveitis developed in 80 (18.4%) children. The cumulative incidence of uveitis according to the International League of Associations for Rheumatology (ILAR) categories is shown in Table 2. There were no uveitis in the systemic nor in the polyarticular RF positive categories, and highest incidence was noted in the juvenile psoriatic arthritis (35.7%) and enthesitis-related arthritis (25.0%) categories. The incidence of uveitis was significantly higher in Finland than in the other Nordic countries (Table 3). Finland also had the highest proportion of children with young age at onset of JIA, but neither gender, oligoarticular onset nor the prevalence of HLA-B27, ANA, or AHA showed any corresponding difference between countries.

**Table 1:** Clinical characteristics at the first study visit in 435 children in the Nordic juvenile idiopathic arthritis (JIA) cohort according to the presence of acute or chronic uveitis during > 7 years of follow-up

	All children		No uveitis		Acute uveitis		Chronic uveitis	
	N		N		N		N	
Female gender	435	286 (65.8)	346	227 (65.6)	9	4 (44.4)	80	55 (68.8)
Age at onset, median (1st, 3rd q)	435	5.5 (2.4, 9.5)	346	6.2 (2.8, 10)	9	10.8 (9.3, 12.9)	80	3.2 (1.8, 5.9)
Oligoarticular onset^a^	435	224 (51.5)	346	180 (52.0)	9	1 (11.1)	80	43 (53.8)
ANA positive	427	116 (27.2)	340	79 (23.2)	9	0 (0)	78	37 (47.4)
AHA >15 U/ml	134	23 (17.2)	112	14 (12.5)	3	1 (33.3)	19	8 (42.1)
RF positive	424	10 (2.4)	336	9 (2.7)	9	1 (11.1)	79	0 (0.0)
HLA-B27 positive	413	87 (21.1)	325	62 (19.1)	9	7 (77.8)	79	18 (22.8)
ESR, median (1st, 3rd q)	354	14 (8, 28)	276	14 (8, 26)	8	23 (10, 41)	70	16 (9, 36)
CRP, median (1st, 3rd q)	350	0 (0, 10)	276	0 (0, 10)	9	0 (0, 16)	65	0 (0, 14)
JADAS27, median (1st, 3rd q)	194	5.1 (2.0, 11.0)	157	5.0 (2.0, 9.8)	7	10.4 (5.6, 14.9)	30	3.8 (1.6, 12.5)
CHAQ, median (1st, 3rd q))	273	0.3 (0.0, 1.0)	222	0.4 (0.0, 1.0)	8	0.4 (0.1, 1.2)	43	0.3 (0.0, 1.0)
DMARD use 2nd visit^b^	350	125 (35.6)	316	111 (35.1)	7	3 (37.5)	27	11 (42.3)

Data are presented as numbers (%), unless otherwise noted

*ANA* antinuclear antibodies measured by immunofluorescence on Hep-2 cells, and *RF* rheumatoid factor; two positive tests taken > 3 months apart in participants with one or more tests taken, *AHA > 15 U/ml* antihistone antibodies IgM/IgG >15 U/ml, *HLA-B27* human leucocyte antigen B27, *Age at onset* age at onset of arthritis in years, *ESR* erythrocyte sedimentation rate, *CRP* C-reactive protein *JADAS27* Juvenile arthritis disease activity score based on 27 joint count, *CHAQ* Child health assessment questionnaire

^a^Oligoarticular JIA 6 months after disease onset, according to the International League of Associations for Rheumatology (ILAR) classification criteria. [25]

^b^Percentage of DMARD = Disease modifying anti-rheumatic drugs including biological agents used within the 2nd visit (median 13 months (IQR 12–14)) in 350 children without uveitis at the time of the 2nd study visit, differentiating into no uveitis, acute, or chronic uveitis during further follow-up

**Table 2:** Presence of acute or chronic uveitis in the different JIA categories according to the International League of Associations for Rheumatology (ILAR) classification criteria at the final study visit in 435 children in the Nordic juvenile idiopathic arthritis (JIA) cohort

JIA category	All children *n* = 435 (% of all)	No uveitis *n* = 346 (%)	Acute uveitis *n* = 9 (%)	Chronic uveitis *n* = 80 (%)	Cumulative total uveitis incidence (95% CI)^a^
Systemic	18 (4.1)	18 (100.0)	0 (0)	0 (0)	0 (0, 18.5)
Oligoarticular persistent	131 (30.1)	106 (80.9)	0 (0)	25 (19.1)	19.1 (12.7, 26.9)
Oligoarticular extended	78 (17.9)	62 (79.5)	0 (0)	16 (20.5)	20.5 (12.2, 31.2)
Polyarticular RF negative	80 (18.4)	62 (77.5)	0 (0)	18 (22.5)	22.5 (13.9, 33.2)
Polyarticular RF positive	3 (0.7)	3 (100.0)	0 (0.0)	0 (0.0)	0 (0, 70.8)
Juvenile psoriatic arthritis	14 (3.2)	9 (64.3)	0 (0.0)	5 (35.7)	35.7 (12.8, 64.9)
Enthesitis-related arthritis	48 (11.0)	36 (75.0)	8 (16.7)	4 (8.3)	25.0 (13.6, 39.6)
Undifferentiated arthritis	63 (14.5)	50 (79.4)	1 (1.6)	12 (19.0)	20.6 (11.5, 32.7)

^a^CI, confidence interval

**Table 3:** Presence of uveitis, antinuclear antibodies, antihistone antibody levels and HLA-B27 according to country of origin in 435 children in the Nordic juvenile idiopathic arthritis (JIA) cohort

	Denmark *n* = 91	Norway *n* = 105	Sweden *n* = 101	Finland *n* = 138
No uveitis	73 (80.2)	86 (81.9)	87 (86.1)	100 (72.5)
Acute uveitis	3 (3.3)	4 (3.8)	1 (1.1)	1 (0.7)
Chronic uveitis	15 (16.5)	15 (14.3)	13 (12.9)	37 (26.8)
Female gender	62 (68.1)	67 (70.5)	67 (66.3)	83 (60.1)
Oligoarticular onset	45 (49.5)	51 (48.6)	55 (54.5)	73 (52.9)
Age at onset	4.8 (2.2, 9.3)	5.9 (2.8, 9.9)	6.9 (3.2, 11.3)	5.1 (2.3, 8.7)
ANA positive	28 (30.8)	32 (32.0)	23 (22.8)	33 (24.4)
HLA-B27 positive	14 (15.6)	29 (27.6)	20 (21.5)	24 (19.2)

Data are numbers (percent) except median (1st and 3rd quartile) for Age at onset; age at onset of arthritis, *ANA* antinuclear antibodies measured by immunofluorescence on Hep-2 cells, two positive tests taken more than 3 months apart in participants with one or more tests takenm *HLA-B27* human leucocyte antigen B27

**Fig. 1:**
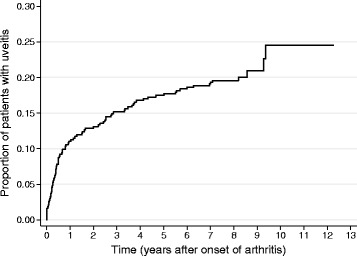
Onset of uveitis in years after onset of arthritis in 435 children in the Nordic JIA cohort, including 5 children developing uveitis before onset of arthritis (time 0)

Time from onset of arthritis to onset of uveitis is shown in Fig. 1, including 5 children in which uveitis was diagnosed before the arthritis, one of these had acute uveitis. Uveitis developed within the first year after onset of arthritis in 48 (53.9%), within 4 years in 73 (82.0%) of the children, and 4 children (4.5%) had onset of uveitis more than 8 years after onset of arthritis, 2 of these had chronic asymptomatic uveitis. The number of children developing uveitis within the first year after onset of arthritis is 6 times the number developing uveitis during the second year, and 16 times the number during the fifth year. All together uveitis developed with an interval of median 0.8 (range − 4.7 to 9.4) years after onset of arthritis, while chronic uveitis developed with a median interval of 0.7 (range − 3.0 to 8.6) years after onset of arthritis.

### Predictors of uveitis

Young age at onset of JIA and ANA were significant predictors of uveitis (Tables 4 and 5). AHA ≥ 15 U/ml was found in 23 (17.2%) and AHA ≥ 30 U/ml in 10 (7.5%) of the 134 participants with AHA analyses. AHA ≥ 30 U/ml was found in 3 of the 5 females with uveitis and AHA analyses. The presence of AHA ≥ 15 U/ml was significantly higher in children with uveitis than in children without (OR 4.8 (1.8 to 13.4)) (Table 4). Serum concentration of AHA IgM/IgG antibodies was significantly higher in the group with uveitis (mean 19.2 U/ml) than in children without uveitis (mean 10.2 U/ml) in the Swedish and Danish cohort with available sera for antihistone analyses (*p* = 0.002; chi-square, data not shown).

**Table 4:** Baseline clinical and demographic predictors of uveitis in 435 participants in the Nordic juvenile idiopathic arthritis (JIA) cohort

	No uveitis		Uveitis		OR (95% CI)
	N	n (%)	N	n (%)	
Female gender	346	227 (65.6)	89	59 (66.3)	1.0 (0.6, 1.6)
Oligoarticular onset	346	168 (48.6)	89	41 (46.1)	0.9 (0.6, 1.4)
Age at onset (< 7 years)	346	189 (54.6)	89	64 (71.9)	2.1 (1.3, 3.5)
ANA positive	340	79 (23.2)	87	37 (42.5)	2.4 (1.5, 4.0)
AHA > 15 U	112	14 (12.5)	22	9 (40.9)	4.8 (1.8, 13.4)
HLA-B27 positive	325	62 (19.1)	87	25 (28.7)	1.7 (1.0, 2.9)
ESR > 20 mm/H	276	95 (34.4)	78	34 (43.6)	1.5 (0.9, 2.5)
CRP > 10 mg/L	276	77 (27.9)	74	27 (36.5)	1.5 (0.9, 2.6)
DMARD use ≤ 2nd visit^a^	316	111 (35.1)	34	14 (41.2)	1.3 (0.6, 2.7)

^a^In 350 children with no uveitis at the time of 2nd study visit, percentage of DMARD used within 2nd visit (median 13 (1st q 12, 3rd q 14) months) in children with no uveitis versus children with uveitis during further follow-up.

Odds ratio (OR) of developing uveitis calculated with logistic regression. Oligoarticular onset, oligoarticular category 6 months after onset of disease, according to the International League of Associations for Rheumatology (ILAR) classification criteria (5); *ANA* antinuclear antibodies measured by immunofluorescence on *Hep-2 cells* two positive tests taken > 3 months apart in participants with one or more tests taken

**Table 5:** Age at onset of arthritis, antihistone antibody levels and presence of antinuclear antibodies according to gender and presence of uveitis in 435 participants in the Nordic juvenile idiopathic arthritis (JIA) cohort

	No uveitis		Uveitis		p
	n	Age at onset of arthritis	n	Age at onset of arthritis	
Female	227	6.8 (2.5, 10.4)	59	3.4 (1.7, 7.5)	< 0.001
Male	121	5.9 (3.2, 9.2)	27	3.9 (2.3, 7.9)	0.390
	n	Presence of ANA	n	Presence of ANA^a^	
Female	222	65 (29.3)	59	27 (45.8)	0.016
Male	118	14 (11.9)	28	10 (35.7)	0.002
	n	AHA IgM/IgG U/ml	n	AHA IgM/IgG U/ml	
Female	72	6.9 (5.2, 11.4)	16	14.2 (7.2, 27.9)	0.003
Male	40	7.1 (4.7, 11.0)	6	8.2 (4.5, 11.4)	0.794

ANA antinuclear antibodies measured by immunofluorescence on Hep-2 cells, two positive tests taken >3 months apart in participants with one or more tests taken. *AHA* antihistone IgM/IgG antibodies U/ml, analyzed only in the Swedish and Danish cohort

^a^numbers (percent), analysed by Chi-square test, otherwise median (1st, 3rd quartile), analyzed with Mann-Whitney rank sum test

Neither gender, oligoarticular onset nor other ILAR categories predicted uveitis. Presence of HLA-B27 was borderline significant as a predictor of uveitis, primarily because there was a high presence of HLA-B27 among children with acute uveitis (Tables 1 and 4). There was no significantly reduced risk of developing uveitis in children that had started disease-modyfing antirheumatic drug (DMARD) treatment, including biologics, neither at the first (data not shown) nor at the second study visit (Tables 1 and 4), taking into account the start date of DMARDs and the date of diagnosis of uveitis. At the final study visit the percentage of children ever using DMARDs was higher in the uveitis group (78.7%) compared to the group without uveitis (53.2%) (data not shown), but the number of children receiving DMARDs to treat primarily their uveitis is not known. These analyses on DMARDs were also performed for methotrexate, showing the same result. There were no significant difference in incidence of uveitis in children that had used etanercept (18.5%) during the disease course versus those that never used etanercept (20.7%), *p* = 0.706. Also the incidence of uveitis was similar in children that had used sulfasalazine (16.1%) versus those that never used sulfasalazine (21.2%), *p* = 0.361. During the disease course, 14.3% of the cohort used sulfasalazine, while 12.4% used etanercept. The main predictors of uveitis in Table 4 were stratified for gender (Table 5). This analysis showed that girls with uveitis had a lower median age at onset of arthritis of 3.4 versus 6.8 in girls without uveitis (*p* < 0.001). The difference in age at onset was highly significant in girls, but not in boys (*p* = 0.390) (Tables 4 and 5). ANA was a significant predictor, both in girls and in boys (Table 4). Analysis stratified for gender showed that median median AHA was significantly higher in girls (*p* = 0.003), but not in boys (*p* = 0.794) (Table 5).

## Discussion

In this Nordic multicenter JIA study, we found a high cumulative incidence of uveitis, and a geographic difference with a higher incidence in Finland compared to Norway, Sweden and Denmark. Young age at onset of arthritis, ANA and AHA were significant predictors of uveitis, but stratified for gender both young age at onset and AHA were significant predictors only in girls. Mean serum levels of AHA were higher in the uveitis group than in the children with no uveitis.

The prospective, longitudinal design and population-based context are the strengths of this multi-center study. The proportion of children lost to follow-up was small compared to other longitudinal studies [26–29]. A weakness is the few patients in each JIA category that made analyses of uveitis and its predictors in some of the categories less reliable. Another weakness is that information on uveitis such as differentiation between acute and chronic uveitis were registered from available ophthalmologic records by the pediatric rheumatologists and not by the ophthalmologists themselves.

The high rate of uveitis in our population is in line with previous findings in the Nordic countries [6, 11, 30, 31]. A prevalence of 8–15% was reported from Sweden [30, 32], 14–18% in northern Norway [11, 31], 9–19% in Denmark [33, 34], while higher rates were previously reported from Finland (24%) [6, 35]. Even though the background rate of HLA-B27 is reported to be higher in northern areas such as Finland and northern Norway [36, 37], we did not find this in our cohort from the Helsinki area in the southern part of Finland. There were also no differences in the presence of ANA between countries. The higher incidence of uveitis during the first 8 years of disease in Finland compared to the other Nordic countries in the present study may be explained by the trend of younger age at onset of arthritis in Finland and/or genetic differences. This finding is in line with the striking worldwide ethnic and genetic differences in uveitis incidence, differences beyond what can be expected by variations in patient accrual and study design. In two large studies from North America of multiethnic populations both Saurenmann et al. and Angeles-Han et al. found that uveitis was more common among Caucasians than among children of black American, Arab and Indian origin [12, 13]. Our cohort was of mostly Caucasian origin.

In line with other studies we found that the risk of developing uveitis during the first year was more than 6 times the risk during the second year, and almost 16-fold the risk during the fifth year after onset of arthritis. This underlines the need for immediate eye examination in children with onset of non-septic or non-specific arthritis even before the diagnosis of JIA is confirmed, and frequent early follow-up when a diagnosis of JIA has been established. Even though a clear majority of 82% developed uveitis within the first 4 years, our data show that uveitis may develop late in the JIA disease course. An important finding was that 4 cases of uveitis developed more than 8 years after onset of arthritis. Of these cases 2 were asymptomatic uveitis, which may not have been diagnosed without long-term screening. Zak et al. found in their 26-years follow-up of a Danish JIA cohort that both new onset of uveitis and complications may develop late [26]. Based on these data long-term ophthalmologic follow-up into adulthood must be considered.

Robust predictors of uveitis will help selecting high-risk children for frequent eye examinations to ensure early diagnosis and treatment. In acute uveitis with symptoms, the diagnosis is easy and predictors not that important, but the differentiation between acute and chronic uveitis is not always clear, and young children may have difficulties expressing complaints of a painful acute uveitis. In line with most publications we have therefore chosen to analyze predictors of all forms of JIA-associated uveitis and compared with children without uveitis [3, 12, 13]. The most consistently reported predictor of uveitis is young age at onset of arthritis. In line with Saurenmann et al. our data confirms that young age at arthritis onset implies high risk of uveitis in girls, but not in boys [38]. The predictors of uveitis are different in girls and boys, even though the rate of uveitis did not differ. This may reflect that different categories of JIA have different types of uveitis with different predicting factors. ANA is another well-documented predictor of uveitis, but this applies only to the immunofluorescence method, and not to the more automatized high-throughput method of ANA ELISA. We were not able to find the gender-specific differences of ANA that Saurenmann et al. found in their Canadian study [38], but there were few ANA positive boys with uveitis in our study.

We found increased risk of uveitis in children with high levels of antihistone antibodies. Previous findings in the Norwegian JIA cohort were now confirmed in 134 Swedish and Danish children, in whom significantly higher baseline serum levels of histone antibodies were found among those who developed uveitis compared to those who did not develop uveitis [11]. In these studies, the histone antibody levels were either borderline positive (≥ 15 U/ml) or positive (≥ 30 U/ml) according to the commercial test, however, the test and the cut-offs are established to detect drug-induced or other types of systemic lupus erythematosus in adults [23]. While the mean serum concentration of combined antihistone IgM and IgG antibodies was 19.2 U/ml in children with uveitis, and 10.2 U/ml in children without uveitis, a mean concentration of 4.3 U/ml was found in healthy children in our previous report utilizing the identical type of AHA EIA kit [11]. The reproducibility of this finding should be tested in other cohorts. Epigenetic modifications of histone such as acetylation and methylation are suggested to be involved in the pathological processes of autoimmunity [18], and may also play a role in the etiology of uveitis [39]. However, no specific intra-ocular antigen has been identified as the target of neither ANA nor AHA, and therefore their potential role in pathophysiology and etiology remains unclear. The presence of ANA immunofluorescence and AHA had similar test properties as predictors of uveitis, but with less predictive ability as the simple measure of young age at onset of arthritis. There is an ongoing discussion on classification in JIA. ANA positive young children are a quite homogeneous group regarding disease characteristics including high risk of uveitis the first 2 years after onset [40, 41]. It is interesting that the higher risk of uveitis in girls with young age at onset, was shown also for girls with higher levels of histone antibodies in our study. Whether young girls with uveitis, presence of ANA and AHA constitutes a biological subset among children with JIA remains to be shown.

We analyzed the onset of uveitis in relation to early DMARD treatment, and found that early DMARD or methotrexate treatment did not seem to prevent later development of uveitis. The inclusion period in our cohort was too short for analyses of prevalence in different time periods, the onset of disease was in the very beginning of the era of biologic agents, and our data are therefore of limited value in clarifying any uveitis-protective potential of specific drugs or modern DMARD treatment in general. Tappeiner et al. have compared prevalence of uveitis in German children with JIA in two time periods up to 2013, and found a lower prevalence in the latest period when DMARDs including biologic agents were more commonly used [42]. Whether DMARDs can prevent or only postpone development of uveitis in JIA is not known.

## Conclusion

We found a high prevalence of uveitis in this Nordic JIA population-based cohort. Significant predictors of uveitis were young age at onset of arthritis and presence of AHA in girls, and presence of ANA in both genders. Further long-term follow-up are needed to focus on prevalence of late-onset uveitis, presence of complications, and impact on quality of life in young adults with JIA-associated uveitis.

## Abbreviations

AHA: Antihistone antibodies
ANA: Antinuclear antibodies
CHAQ: Childhood Health Assessment Questionnaire
CI: Confidence interval
CRP: C-reactive protein
DMARD: Disease-modyfing antirheumatic drugs
EIA: Enzyme Induced Assay
ERA: enthesitis-related arthritis
ESR: Erythrocyte sedimentation rate
HLA-B27: Human leucocyte antigen B27
ILAR: International League of Associations for Rheumatology
IQR: Interquartile range
JADAS27: Juvenile Arthritis Disease Activity Score
JIA: Juvenile idiopathic arthritis
OR: Odds ratio
RF: Rheumatoid factor
SUN: Standardization of Uveitis Nomenclature
